# Community experiences and perceptions of reproductive health vouchers in Kenya

**DOI:** 10.1186/1471-2458-13-660

**Published:** 2013-07-16

**Authors:** Rebecca Njuki, Francis Obare, Charlotte Warren, Timothy Abuya, Jerry Okal, Wilson Mukuna, Lucy Kanya, Ian Askew, Piet Bracke, Ben Bellows

**Affiliations:** 1 Population Council, P.O. Box 17643-00200, Nairobi, Kenya; 2 Moi University, P.O. BOX 3900, Eldoret, Kenya; 3 Ghent University, B-9000, Ghent, Belgium

## Abstract

**Background:**

Research on demand-side health care financing approaches such as output-based aid (OBA) programs have focused on evaluating the role of the programs improving such outcomes as utilization of services and quality of services with limited focus on the experiences and perceptions of the target communities. This paper examines community members’ views of the output-based aid voucher program in Kenya.

**Methods:**

A household survey was conducted in 2010 among 1,336 women aged 15-49 years living in the catchment areas of contracted health facilities in three districts participating in the voucher program (Kisumu, Kiambu and Kitui). Twenty seven focus group discussions were conducted with voucher users, non-users, opinion leaders and voucher distributors in the three districts as well as in Nairobi. Analysis of the quantitative data involved frequency distributions and cross-tabulations. Qualitative data were transcribed and analyzed by adopting framework analysis and further triangulation of themes across respondents.

**Results:**

Majority (84%) of survey respondents had heard about the safe motherhood voucher compared to 24% and 1% that had heard about the family planning and gender-based violence recovery services (GBVRS) vouchers respectively. Similarly, 20% of the respondents had used the safe motherhood voucher compared to 2% for family planning and none for the GBVRS vouchers. From the community members’ perspectives, the voucher program is associated with improvements in access to health services for poor women, improved quality of care, and empowerment of women to make health care decisions. However, community members cited difficulties in accessing some accredited health facilities, limitations with the system of selling vouchers, lack of male involvement in women’s reproductive health issues, and poor understanding of the benefits associated with purchasing the voucher.

**Conclusion:**

The findings of this paper showed that the voucher program in Kenya is viewed by the community members as a feasible system for increasing service utilization, improving quality of care, and reducing financial barriers to accessing reproductive health services. However, the techniques of program execution such as proper information and availability of the distributors as well as local attitudes influence whether vouchers are purchased and used.

## Background

Many poor women face huge financial barriers that limit their access to quality maternal and reproductive health care [1]. Out-of-pocket costs arising from birth complications can be prohibitive or catastrophic, thereby excluding many mothers from receiving necessary care or pushing families further into poverty [2]. Experience has shown that routine financing of health inputs such as staff costs, equipment and buildings without associating these inputs with the services (outputs) delivered does not necessarily enable access to quality services among the poor [3-5]. To overcome this challenge, alternative financing mechanisms have been designed that transfer purchasing power to the consumer of health services, thereby motivating providers to offer more accessible and higher quality services so that they can attract and serve consumers who purchase their services [6-8].

One such financing mechanism is the output-based aid (OBA), which targets the poor and under-served populations with subsidized essential health services [5,9]. In OBA voucher programs, a voucher management agency (VMA) distributes or sells vouchers for specific services at a subsidized price to intended beneficiaries, and then reimburses healthcare facilities for providing services to clients presenting with vouchers. OBA programs subsidize specific health care packages based on provision of care with pre-defined quality standards [6,10,11]. Most OBA programs have the following goals: to improve service quality, to stimulate client use of selected services, to target services among high-priority populations where service uptake is low in the absence of the subsidy, and to contain costs [12-16]. Most OBA programs have a supervisory or regulatory body that meets periodically to oversee their operations including contracts with providers. OBA programs invite service providers (public, non-profit or private-for-profit) to participate. Those agreeing to participate can only do so if they demonstrate service provision at a specified standard of quality of care. Usually a number of providers are accredited to create competition and give consumers choice.

In Kenya, the reproductive health (RH) vouchers program has been implemented by the Government since 2006 with major funding from the German Development Bank (KfW). Details of the implementation process are discussed elsewhere [17]. The program aims to improve utilization of selected services among economically disadvantaged women of reproductive age (15-49 years) in four districts (Kitui, Kiambu, Kilifi, and Kisumu), and two informal settlements in Nairobi (Korogocho and Viwandani). The program offers three packages of reproductive health care services: (1) safe motherhood (SM) including up to four antenatal care visits, delivery (normal or Caesarean) and complications as well as one postnatal care visit within six weeks post-delivery; (2) long-term family planning (FP) methods (implants, intra-uterine contraceptive device [IUCD], and voluntary tubal ligation); and (3) gender-based violence recovery services (GBVRS) including medical examination, treatment and counseling.

Potential beneficiaries for the SM and FP vouchers are identified in the community by voucher distributors appointed by the VMA using a poverty grading tool consisting of eight items including housing, access to health, water sources, sanitation, daily income and meals per day. Those scoring between 8 and 16 points on the poverty grading tool qualify to purchase the vouchers from the distributors at a subsidized price equivalent to US $2.50 for SM and US $1.25 for FP vouchers. They then redeem the vouchers for services at accredited facilities that comprise public, private-for-profit and private-not-for-profit providers. The GBVRS voucher is, on the other hand, freely available at accredited facilities to all those who are in need of the services regardless of socio-economic status; this voucher functions primarily as a means for the provider to recover the costs of the GBVR services and for ensuring that quality of care is maintained through accreditation. The distributors are, however, expected to sensitize community members about the availability of the voucher.

The voucher program was conceptualized in the context of poor reproductive health indicators in the country. For instance, at the time of the program’s inception, the maternal mortality ratio was 414 deaths per 100,000 live births while infant mortality rate was 77 deaths per 1,000 live births [18]. In addition, although 88% of expectant women received antenatal care from a trained service provider, only 40% of the births were delivered in a health facility while 42% of the births were delivered under the supervision of a health professional [18]. Moreover, births to women from the poorest households were more than four times less likely to be delivered in a health facility or under skilled care compared to those to women from the richest households (16% and 74% respectively for health facility delivery and 17% and 74% respectively for skilled delivery care, [18]. In addition, the contraceptive prevalence rate had stagnated at 39% since 1998, unmet need for family planning was 25% while women desired fewer children than they actually gave birth to (average of 3.9 children desired compared to the total fertility rate of 4.9 children per woman [18].

Several evaluations of output-based financing schemes for RH services have demonstrated some positive effects of the programs with respect to increased use of skilled birth attendance, hospital deliveries, antenatal care, family planning, health counseling, and sexually transmitted infections (STI) services as well as reduction of inequality to health care access, improvements in quality of services, and reduction in out-of-pocket expenditure [10,15,16,19-23]. There has, however, been little documentation of community perceptions of and experiences with accessing and using reproductive health vouchers. In addition, although studies of voucher programs generally find associations with improved outcomes, larger contextual issues and the experiences of potential or actual beneficiaries are often unexplored. This paper uses a mixed-methods approach to examine community experiences and perceptions of the OBA voucher program in Kenya. Understanding the community needs and preferences in the context of health care interventions enables the alignment of policy and programs to public expectations and can thus determine the success of the programs.

## Methods

This paper uses quantitative and qualitative data that was collected in 2010 in three voucher sites: Kitui, Kiambu and Kisumu districts.

### Household survey

The household survey was conducted among 1,336 women aged 15-49 years living within a five-kilometer radius of health facilities offering services for voucher clients in Kitui, Kiambu and Kisumu districts. At least 400 women were targeted in each district in order to detect statistically significant differences in key reproductive health indicators at 95% confidence level and 80% power. A multi-stage sampling design was used to identify study participants. In the first stage, 14 sub-locations (the smallest administrative unit in Kenya) were randomly sampled in each district. In the next stage, three enumeration areas were randomly selected from each sub-location. The local administration then assisted with the identifying households that were considered poor in the enumeration area for inclusion in the study. The identified households were screened using the poverty grading tool that the voucher management agency uses to identify beneficiaries in order to capture as many individuals who would qualify for the vouchers as possible. In each district, the target was 75% poor (those who would qualify for vouchers) and 25% non-poor women for comparison.

In each household, one female member who gave birth in the past 12 months or was pregnant at the time of the survey was interviewed. For households with more than one member satisfying this criterion, the youngest was interviewed since they are more likely to experience poor reproductive health outcomes compared to older women. In case, the selected household did not have a female member who gave birth in the past 12 months or was pregnant, any willing female member aged 15-49 years was interviewed. Respondents were asked about their background characteristics (such as age, education level, and marital status), use of reproductive health services, and awareness, perceptions and use of the reproductive health vouchers. Table 1 presents the distribution of respondents in the three study sites by background characteristics. The majority of the respondents were aged 25-34 years, had primary level education, were from rural areas, had stayed at the place of residence for more than five years, and were married or living with a man at the time of the survey.

**Table 1:** Percent distribution of participants in the household survey by background characteristics according to study site

	**Kitui (%)**	**Kiambu (%)**	**Kisumu (%)**	**All sites (%)**
	**(%)**	**(%)**	**(%)**	**(%)**
**Characteristics**	***N*** **= 517**	***N*** **= 411**	***N*** **= 408**	***N*** **= 1,336**
Age group (years)				
15-24	26.3	28.5	49.7	34.1
25-34	42.6	51.1	39.0	44.1
35-44	26.5	18.5	9.1	18.7
45 and above	4.6	1.9	2.2	3.1
Highest education level				
No schooling/pre-unit	5.0	0.5	1.2	2.5
Primary	77.0	56.2	75.3	70.1
Secondary and above	18.0	43.3	23.5	27.5
Place of residence				
Urban	7.9	28.0	29.2	20.6
Rural	92.1	72.0	70.8	79.4
Duration of residence				
<5 years	23.6	37.0	46.8	34.8
5 or more years	66.3	54.5	48.0	57.1
Always	10.1	8.5	5.2	8.1
Marital status				
Never married	12.2	13.6	11.0	12.3
Married/living together	76.8	79.1	79.9	78.4
Formerly married^a^	11.0	7.3	9.1	9.3
Poverty status^b^				
Poor	86.3	62.0	20.1	23.1
Non-poor	13.7	38.0	79.9	76.9

^a^Separated, divorced or widowed; ^b^According to the criterion used by the voucher management agency to identify beneficiaries.

Written informed consent was obtained from all participants before conducting the interviews. For respondents who could not read and write, the interviewers read the written consent form for them before they could append their thumb prints. Information was captured using personal data assistants (PDAs). Interviews were conducted in English, *Kiswahili* (the national language) or the local languages. A full description of the household survey design has been published elsewhere [20]. The data from the PDAs were downloaded into Access database and exported into Stata 10 for analysis using frequency distributions and cross-tabulations.

### Qualitative interviews

A total of 27 focus group discussions (FGDs) were conducted in 2010 with groups of female voucher users and non-users aged 18 years and above, community leaders and voucher distributors who are residents of the regions and are recruited by the VMA. A total of 178 respondents participated in the FGDs. The FGDs for voucher users and non-users were exclusive for women and comprised separate groups for participants younger than 25 years and those aged 25 years or older. FGDs with voucher distributors and community gatekeepers were mixed groups of men and women. All voucher distributors and community gatekeepers were eligible for recruitment and were not distributed into any group. The selection of FGD participants was done through joint community mobilization that was conducted by the research assistants and field supervisors with the assistance of the VMA field managers, community elders and community health workers. Table 2 presents the distribution and composition of FGD participants.

**Table 2:** Distribution and composition of participants in the focused group discussions

**Participant group**	**Composition**	**Number of FGDs**	**Number of participants**
Voucher users	Women who were currently or had been voucher users (<25 years)	4	24
	Women who were currently or had been voucher users (25 years and over)	4	27
Voucher non-users	Women who had never used voucher (<25 years)	5	37
	Women who had never used vouchers (25 years and over)	5	35
Local community gatekeepers	Mixed group FGD with local chiefs who acted as community gatekeepers and opinion leaders	5	30
Voucher distributors	Voucher distributors employed by VMA	4	25
Total		27	178

FGD: Focus group discussion; VMA: Voucher management agency.

The voucher non-users were also screened for eligibility using the poverty grading tool. The discussions sought to gain a deeper understanding of the motivations, perceptions, and priorities of the local communities regarding health services in general and vouchers in particular. Specifically, the FGDs addressed the following broad themes: (i) motivations for seeking healthcare and selection of RH services, (ii) attitudes towards the voucher program, (iii) quality of care, and (iv) contraceptive and sexual health behavior, including communication with the partner and other community members about accessing RH services.

The FGDs consisted of between six and eight participants with discussions lasting one to two hours. Each FGD was conducted by two trained research assistants—a facilitator and a note-taker. Informed consent was obtained from all participants before the discussions. The discussions were tape-recorded in local languages and then transcribed into Word format and translated to English. There was, however, no back-translation of the transcripts into the local languages. The transcribed texts were then transferred to NVIVO 8 analysis software and analyzed by two researchers. Following coding, a full list of themes was available for categorization within a hierarchical framework of main and sub-themes. The thematic framework was then systematically applied to all of the interview transcripts. Patterns and associations of the themes were identified and compared and contrasted within and between the different groups of respondents.

Ethical approval for conducting the study was granted by the Population Council’s Institutional Review Board and the Kenya Medical Research Institute Ethical Review Committee.

## Results

### Awareness about vouchers

Results from the household survey show that the safe motherhood voucher was the most well known while the GBVRS voucher was the least known. In particular, 84% of the women interviewed reported having heard about the SM voucher, 24% had heard about the FP voucher while only 1% had heard about the GBVRS voucher (Table 3). Findings from the FGDs confirmed these results. Nonetheless, despite the high levels of awareness about the SM voucher, its benefit package was not well understood even among voucher users. For example, some participants thought that the SM voucher was only for use during delivery or in cases of emergency. Moreover, there was lack of understanding of whether one could obtain both SM and FP vouchers:

*“I have only heard of the family planning and safe motherhood voucher, the one for rape (GBV) I have heard of them today here”.* (FGD voucher user)

*“In a family, if someone is pregnant, people will encourage you; they even contribute to buy that card so that in case of an emergency you are taken to the hospital to get assistance”.* (FGD voucher user)

“*We in interior areas have not obtained the vouchers, most of us come from far to visit the nearest hospital, there is lack of money and the voucher distributors are few. We are also not given information about vouchers adequately by the distributors. Some do not know about the vouchers, they are not enlightened. They should put more effort so that the voucher distributors are many and to be brought near”.* (FGD voucher non-user)

*“Educate people because many have not heard about the vouchers. Educate them in the barazas [public gatherings] at the chief’s place by bringing educators”.* (FGD voucher non-users)

**Table 3:** Percent distribution of survey participants by awareness and use of reproductive health services and vouchers according to study site

**Awareness and use of vouchers**	**Kitui (%)**	**Kiambu (%)**	**Kisumu (%)**	**All sites (%)**
	***N*** **= 517**	***N*** **= 411**	***N*** **= 408**	***N*** **= 1,336**
Awareness and use of family planning				
Ever heard of family planning	94.6	98.8	98.0	96.9
Ever used family planning	45.8	85.6	65.9	64.2
Currently using family planning	26.1	61.8	45.3	43.0
Ever heard of the vouchers				
Family planning voucher	21.1	33.1	16.9	23.5
Safe motherhood voucher	87.8	82.5	79.4	83.6
Gender-based violence recovery	0.4	2.0	2.0	1.4
Ever used the vouchers				
Family planning voucher	1.0	5.4	1.0	2.3
Safe motherhood voucher	21.3	17.8	20.8	20.1
Either type of voucher	21.7	19.2	21.3	20.8

#### Utilization of vouchers

Only two percent of the women interviewed in the household survey had ever used the family planning voucher while 20% had used the safe motherhood voucher, with 21% having used either of the two types of vouchers (Table 3); none of the women reported having used the GBVRS voucher. Program data show that as of March 2011, a total of 139,085 safe motherhood vouchers had been sold of which 95,356 (69%) had been redeemed for services [24]. Similarly, 52,533 family planning vouchers had been sold of which 30,246 (58%) had been redeemed while 780 gender-based violence recovery services vouchers had been used over the same period [24].

The use of the SM voucher also featured prominently in the FGDs compared to other vouchers as exemplified by the following quotes:

*“I have two daughters and I used the SM voucher during their delivery, when I feel that I’m almost going into labor I buy the SM voucher card to use during delivery”.* (FGD voucher user)

*“During pregnancy I used to go to the clinic at xxxx and it was free. I didn’t have the money to pay for ultrasound. The voucher catered for it and I was educated on many things. I was given all the services including ultra sound because they suspected I had twins. They verified I only had one child”.* (FGD voucher user)

*“Many women were not able to go and deliver in the hospital, but when they get the voucher they now deliver at health facilities. We have heard cases where you ask a mother how many kids do you have and she says may be five and they were all delivered at home but now when they have the voucher they go to deliver in the hospital”.* (FGD Distributors)

*“I heard if you don’t have the delivery one you can’t buy the family planning one”.* (FGD voucher non-user)

*“Some mothers say the family planning makes women to become less attractive to the husband and when a woman becomes less interested in sex, the man may opt to go and look for other women. Therefore some husbands refuse to practice family planning methods”.* (FGD voucher non-users)

### Perceived benefits of using vouchers

**Figure 1:**
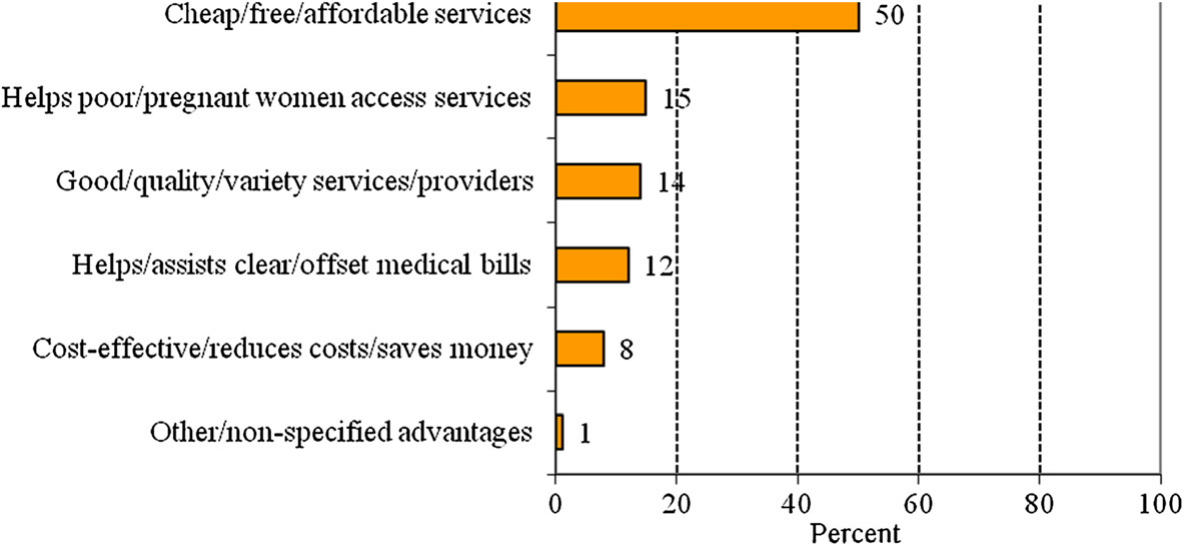
Percent distribution of women from the household survey who would recommend the use of vouchers to a friend by the major reasons (N = 276).

Nearly all women (99%) in the household survey who had ever used any of the reproductive health vouchers indicated that they would recommend its use to a friend with no significant variations by background characteristics. The most commonly cited reasons for recommending the voucher were that it: (i) caters for cheap or affordable services; (ii) helps poor or pregnant women access services; (iii) enables one to obtain good quality or variety of services from a range of providers; and (iv) helps to clear or offset medical bills (Figure 1).

FGD participants noted that the voucher program not only improved access to essential reproductive health services for poor women but also enhanced the quality of services they received. For example, participants noted that the program protected women from detention in health facilities for failure to pay for medical services and from abandonment by health workers for those who cannot afford to pay for delivery services. The majority of voucher users also reported that they received better and prompt services from accredited facilities compared to previous instances when they sought services without a voucher. They further noted that accredited facilities had improved the comfort of their clients through improved meals, provision of warm bathing water, hot drinks and increased number of beds. Other participants reported that the voucher provided an opportunity for improved communication with providers who offered better health education and counseling than before:

*“When you hold a voucher, you have no stress of lack of money; therefore if I am pregnant and I am due for delivery I just head to the hospital because I have a voucher. I go early to the hospital, I’m never late”.* (FGD voucher user)

*“I also delivered at [name of facility] and from what we knew, [name of facility] was for the rich. Even those who heard that I delivered at [name of facility] said I was blessed. I’m grateful because I never would have gone to [name of facility], the voucher has taken us to another level”.* (FGD voucher user)

*“When you go to the hospital, you are treated well, you are fed nicely, you are given water to bathe, I mean it’s all nice, it’s like they receive something after you get assisted. You are treated very well, it’s not like before. His [the health provider’s] attention is there and he’s very busy asking you what’s wrong and he wants to serve you well”.* (FGD voucher user)

*“Communication has really changed; unlike before you would be advised to see a senior doctor and this would have been really hard. At the moment it is easy. Doctors are keen to listen after you explain they try as hard to assist. Before, the doctors were not concerned about patients like today. When you have the voucher card you receive good service”.* (FGD voucher user)

Besides improved access to and quality of services, FGD participants noted that the voucher program has contributed to reductions in home deliveries, maternal and newborn deaths, and malnutrition by empowering women to take charge of their health care decisions. Participants reported that the voucher empowered women in family planning by providing an opportunity to use long-term methods that were otherwise not affordable. They further noted that the voucher provided women with the financial support men denied them during childbirth and in using family planning methods:

*“Maternal and neonatal deaths have been reduced and there are many cases where one falls ill after delivery, since you have the voucher you just head back to the facility and you are taken care of by the health worker”.* (FGD voucher user)

“*The family planning has also helped because women who didn’t have money can now go for the Norplant or choose the method they want and they can have the chance to plan their families”.* (FGD voucher user)

*“If your husband cannot take care of you during pregnancy the voucher will cater for your bills. Some men even run away when you get labor pains”.* (FGD voucher user)

### Perceived challenges of using the vouchers

Only two respondents in the household survey who had ever used the voucher indicated that they would not recommend its use to a friend, mainly because it is not free and that providers were not friendly. Among FGD participants, poor quality services were only reported by those from one site where voucher users reported discriminatory treatment and felt that priority was given to those who pay cash. Consequently, women who had already purchased the voucher preferred to pay cash or did not use the voucher because they had experienced discrimination at the facility. There were also perceptions that voucher users were being subjected to unnecessary Caesarean section deliveries in some facilities. The following excerpts highlight some of these perceived challenges:

“*The reception when you go to deliver is bad. Once they see the voucher, you are not lucky. There is a problem there because you cannot be received the same way as a person who has money. You have to wait until they serve those with money and at times you go back home with your problems because maybe it’s late and the doctor has left. They take the voucher holders lightly”.* (FGD voucher user)

*“Some women were saying that if you have the voucher, since the services are free, you are treated badly and with a lot of arrogance, so some want them, others don’t.”* (FGD voucher non-user)

*“People prefer paying on their own rather than take the voucher because of the problems you go through getting the voucher and when you go to the hospitals”.* (FGD voucher non-user)

“*Some fear the voucher because in most facilities like in xxxx, most people are taken for operation if they have the voucher”.* (FGD voucher user)

*“Some of the potential clients believe that when they purchase the voucher, the will all go through Cesarean delivery so they don’t purchase the voucher”.* (FGD distributor)

Another challenge that emerged from the FGDs was the poor road infrastructure and high transport costs in remote areas, which make the distribution of vouchers and access to the accredited facilities difficult. In some settings, the transport costs to accredited facilities were higher than the service costs in more accessible non-accredited facilities:

*“For us … we would like an accredited hospital nearby. Even if you have a voucher, the taxi charges about 3000 shillings [equivalent to US $35.70] to hospital, you would rather pay the 1,200 shillings [equivalent to US $14.25] they charge for delivery here (in a non-accredited facility) rather than take a taxi”.* (FGD voucher user)

“*Transport is a problem due to inaccessible roads; a woman might be far and without means of transport but since she has a voucher card an ambulance can be sent to pick her from her home but the roads are inaccessible”.* (FGD chiefs)

Voucher distributors, voucher users and opinion leaders felt that the poverty grading tool sometimes left out genuine cases. For example in Kitui, households with shelters made from brick (the only material available) but had a thatched roof and earth floor were scored as rich. In addition, there were claims that some women falsified personal information in order to be considered eligible. Voucher distributors further reported that women who lacked national identity cards failed to obtain vouchers when the program started. Although parental/guardian identification cards were later allowed, it discouraged adolescent girls who needed to purchase family planning vouchers without disclosing the information:

“*You find also in the community, there are so many mothers but they do not have identity cards, so they have the money to purchase the voucher but they do not have identity cards required to buy vouchers”.* (FGD voucher distributor)

*“Another challenge is that there is another group that is sidelined, for instance, they normally asked for the husband’s or the mother’s identity card. So there are some cases of single mothers or young mothers who are denied the voucher”.* (FGD Voucher user)

*“To get him will be a problem because you may find he has gone to see another client and you will not get him. We need to increase the distributors and they need to have fixed places and time to get them”.* (FGD voucher user)

Findings from the FGDs further indicate that negative male attitudes towards the voucher and lack of support during childbirth discouraged some eligible women from purchasing the voucher while others who had purchased the voucher were reluctant to use it. Participants noted that some men opposed the use of family planning methods in general or long-term methods in particular while some women believed it negatively impacted on their sexual attractiveness. Women in such relationships risked being physically abused by their spouses if found using the methods. It was further noted that many men have not accepted the vouchers because the program targets the very poor and they feel that their stature as household heads is demeaned if their wives purchase the voucher:

*“In this area, most young men have gone into alcohol…And you as the woman, you have been left with all the responsibilities. Even when I decided to get the voucher, I didn’t tell him”.* (FGD voucher user)

“*They don’t take the vouchers. If you ask them they say that they are for the poor…So if she takes, the family will be laughed at because they are poor. So even if you give one, she cannot tell the husband about the voucher”.* (FGD voucher distributor)

“*Most of the mothers have not used the long-term methods, we are trying to encourage them, we are convincing them, we are giving them the advantages of long-term methods but yes there are some misconceptions about FP methods. Others are saying no, they cannot use that method, so we have to talk to them from time to time and some have accepted. Basically concerning FP, they have misconceptions, for example if a lady undergoes something like BTL [Bilateral Tubal Ligation], she says she’s becoming cold; another says that if she undergoes BTL, she will become pregnant. So you find that the uptake of SMH (Safe Motherhood) is high than for FP”.* (FGD voucher distributors)

The fear of HIV testing and concerns about confidentiality of test results also emerged in the FGDs as a possible challenge for the uptake of the vouchers. Participants reported that the use of the voucher may be associated with mandatory HIV testing that forms part of antenatal and delivery care and the fear of knowing the sero-status of the mother or the spouse. The preference to deliver at home or with traditional birth attendant (TBA) was partly attributed to the fear of being tested for HIV at the health facilities.

*“Some of them fear going for medical test like HIV /AIDS, so they prefer going for TBA’s (traditional birth attendants) where there are no tests done. They fear knowing their status”.* (FGD chief)

*“Some women take the voucher but they don’t want to go to hospital because they believe they will be tested for HIV/AIDS. They know that if they go they will be tested; they are afraid of being tested and knowing their status. Some women after being tested for HIV and are found positive opt never to go to health facilities for good”.* (FGD voucher user)

“*The most pressing reproductive health issues for women here is that they fear HIV testing. Some women fail to go back to the hospital after the test”.* (FGD voucher non-users)

## Discussion

This paper examined the community experiences with and perceptions of the reproductive health vouchers program in Kenya. One major finding is that there were wide variations in awareness and use of the various types of vouchers, that is, safe motherhood, family planning and gender-based violence recovery services. In particular, the safe motherhood voucher was the most well known and widely used while the gender-based violence recovery services voucher was the least known and used. These variations could partly be attributed to the distribution system whereby the safe motherhood voucher and family planning vouchers are distributed in the community while the GBVRS voucher is only available at the facilities accredited to offer the services. Besides, cultural factors and stigma associated with gender-based violence impede the uptake of such services [15,25-27]. The lower awareness and utilization of family planning compared to safe motherhood voucher is consistent with the low awareness and use of long-acting family planning methods in the country, negative male perceptions and societal misconceptions about family planning as well as perceptions of poor treatment of clients by providers [15,25,26].

Second, community members generally perceived the voucher program as beneficial, in that it has led to improvements in access to essential reproductive health services for poor women as well as in the quality of services provided. There was also a general perception that the voucher program has contributed to reductions in home deliveries, maternal and newborn deaths, and malnutrition by empowering women to take charge of their health care decisions. Some community members identified challenges to the uptake of vouchers, such as availability of the distributors, discrimination against voucher clients in favor of paying clients in some facilities, difficulties in reaching accredited health facilities from remote areas, perceptions that women may be subjected to an unnecessary Caesarean section, lack of men’s involvement and support towards women’s reproductive health issues, and fear of HIV testing. Another challenge arose from the fact that some women did not fully understand the benefit package associated with the vouchers, especially the SM voucher, thinking that it could only be used for pregnancy-related complications. This raises questions as to the kind of information that the distributors give voucher clients.

These challenges could be addressed through improved marketing of the services that are subsidized by the voucher program to ensure correct understanding of the package and to address negative perceptions; innovative approaches to strengthen community engagement, especially among men; periodically assessing the eligibility criteria to ensure the poorest are not excluded; and conducting a costing study to determine the potential for subsidizing transport costs in the voucher program. Strengthening community engagement can also ensure that women are always aware of when and where the distributors are available. Some of these strategies have been employed by voucher programs elsewhere. For example, voucher programs in Bangladesh and Cambodia cover transport costs and reimburse care givers in public facilities to motivate them [15,26]. Moreover, both countries have successfully partnered with the recipient communities to improve targeting of the poor [15,25,26].

The findings of this paper may be influenced by the study’s limitations. First, given that FGD participants were not randomly selected, their views may not representative of the opinions of the general population or of all voucher users (for FGDs involving voucher users). It could be that those who were approached and agreed to participate in the discussions had strong views about reproductive health care in general and the voucher program in particular. However, the fact that some of the qualitative findings—especially regarding awareness and use of vouchers—are consistent with findings from the household survey suggest that the bias attributable to the nature of the sample of participants in FGDs may have been minimal. Second, FGD sessions may have been dominated by the opinions of a few thereby biasing the findings. However, apart from being trained to undertake the study, the facilitators were individuals with basic training in qualitative research and therefore understood the importance of ensuring that the discussions were balanced. Third, societal attitudes and beliefs may not only affect the uptake of family planning and gender-based violence recovery services but also determine the extent to which these issues are openly discussed in a FGD forum. Fourth, due to financial and time constraints, there was no back-translation of the transcripts to local languages to determine if some meanings may have been lost in the process of translation to English.

## Conclusions

Despite the above limitations, the findings of this paper show that the voucher program in Kenya has been successful in providing services; improving access and the quality of care provided to poor women in target areas. Efforts to improve the efficiency and effectiveness of the program need to consider enhancing linkages among all the actors including the voucher management agency, the targeted communities, potential voucher users, and the contracted health facilities. Standardizing the information package on the program for all actors will help address the emerging contextual factors that indirectly affect its functioning. Further, the targeting mechanism needs to be strengthened alongside accreditation of more health facilities. The program should also consider introducing transport facilitation allowance as part of the benefit package.

## Abbreviations

CHW: Community health worker; FGD: Focus group discussions; FP: Family planning; GBVRS: Gender-based violence recovery services; OBA: Output-Based Aid; PDA: Personal data assistant; SM: Safe motherhood; VMA: Voucher management agency.

## Competing interests

The authors declare that they have no competing interests.

## Authors’ contributions

RN: Involved in data collection, data analysis, drafting, re-organizing and overall revision of the manuscript. FO: Involved in data collection, data analysis, drafting, re-organizing and revision of the manuscript. WM: Was involved in data analysis and revision of the manuscript. CW: Conceptual design of the study and revision of the manuscript. TA: Involved in the revision of the manuscript. JO: Involved in the revision of the manuscript. LK: Involved in the revision of the manuscript. IA: Conceptual design of the study and revision of the manuscript. PB: Involved in the revision of the manuscript. BB: Involved in the revision of the manuscript. All authors read and approve the final manuscript.

## Pre-publication history

The pre-publication history for this paper can be accessed here:

http://www.biomedcentral.com/1471-2458/13/660/prepub

## Adherence to RATS guidelines

We confirm that the paper adheres to RATS guidelines for reporting qualitative studies as outlined in *Qualitative Research Review Guidelines–RATS* available at: http://www.biomedcentral.com/ifora/rats. In particular, the research question, the study design, the criteria for selecting participants, and the analytical approach are explicitly described.
